# Clinical applications of custom 3D printed implants in complex lower extremity reconstruction

**DOI:** 10.1186/s41205-020-00083-4

**Published:** 2020-10-02

**Authors:** Rishin J. Kadakia, Colleen M. Wixted, Nicholas B. Allen, Andrew E. Hanselman, Samuel B. Adams

**Affiliations:** Department of Orthopaedic Surgery, Duke University, 4709 Creekstone Drive, Suite 300, Durham, NC 27703 USA

**Keywords:** Implants, Foot and ankle, Orthopaedic surgery, Surgery, Instrumentation

## Abstract

**Background:**

Three dimensional printing has greatly advanced over the past decade and has made an impact in several industries. Within the field of orthopaedic surgery, this technology has vastly improved education and advanced patient care by providing innovating tools to complex clinical problems. Anatomic models are frequently used for physician education and preoperative planning, and custom instrumentation can assist in complex surgical cases. Foot and ankle reconstruction is often complicated by multiplanar deformity and bone loss. 3D printing technology offers solutions to these complex cases with customized implants that conform to anatomy and patient specific instrumentation that enables precise deformity correction.

**Case presentation:**

The authors present four cases of complex lower extremity reconstruction involving segmental bone loss and deformity – failed total ankle arthroplasty, talus avascular necrosis, ballistic trauma, and nonunion of a tibial osteotomy. Traditional operative management is challenging in these cases and there are high complication rates. Each case presents a unique clinical scenario for which 3D printing technology allows for innovative solutions.

**Conclusions:**

3D printing is becoming more widespread within orthopaedic surgery. This technology provides surgeons with tools to better tackle some of the more challenging clinical cases especially within the field of foot and ankle surgery.

## Introduction

The use of 3D printing has revolutionized the manufacturing process across various industries and enabled the creation of precise customized products. The origin of this technology can be traced back to 1984 when Charles Hull filed a patent for the stereolithography fabrication system and eventually began selling 3D printers for commercial use in 1988 [1, 2]. This technology has drastically changed over the years and is currently being employed in almost every major manufacturing sector. Three dimensional printing technology has recently been more utilized in medicine and specifically in the field of orthopaedic surgery. Within orthopaedic surgery, 3D printing has allowed for the development of anatomical models that can be used for preoperative planning and education and more revolutionary, the development of patient specific instruments and implants that can be used intraoperatively. This technology can be helpful in cases of complex lower extremity reconstruction as deformity and bony defects can be challenging to manage. The ability to customize surgical instruments and implants to match the complex three dimensional deformity that is frequently seen with foot and ankle pathology has made 3D printing a novel tool when tackling these challenging problems. The applications of 3D printing within foot and ankle surgery are endless and as the technology continues to progress, the clinical utility will become more evident.

## 3D printing within orthopaedic surgery

3D printing technology is already being utilized within other subspecialties in orthopaedic surgery. Takeyasu et al. reported on a series of 30 patients who underwent correction of cubitus varus deformity – a complex deformity of the elbow - with custom made 3D printed surgical guides. They found statistically significant improvements in alignment and 90% of patients reported excellent results [3]. For total knee arthroplasty (TKA) and total hip arthroplasty (THA) cases in patients with complex or unique anatomy, 3D printed patient specific instrumentation and implants have become a viable alternative. Compared to standard implants, patients with custom implants reported fewer adverse events, decreased intraoperative blood loss, and were less likely to be discharged to an acute care facility or rehabilitation center in a recently published study [4]. 3D printing technology has also allowed engineers to improve upon standard implant designs through the manufacturing process. Patients who underwent revision hip arthroplasty with 3D printed acetabular cups demonstrated improved stability, better hip scores, and decreased pain [5]. While a majority of the products of 3D printing technology provides direct patient benefit, surgical trainees can develop, practice, and refine their technical skills with realistic 3D patient models as well. A survey of resident surgeons regarding the clinical utility of 3D models of posterior column fractures reported high overall satisfaction with these models when planning their surgical approach [6]. There are many applications of 3D printing already in place within orthopaedic surgery and the applications will continue to grow as technology advances and access to 3D printers improves.

## 3D printing in foot and ankle surgery

Foot and ankle pathology can be challenging to manage given the complexity of the three dimensional anatomy and interactions between the several articulations. Deformity correction requires an appreciation for normal anatomy but also an understanding of the deformity in multiple planes. 3D printing technology can assist in the preoperative planning of these complex cases by providing precise anatomical models to plan out hardware placement and osteotomies. Jastifer et al. reported on using a 3D model to help plan for deformity correction for an ankle fracture malunion. The authors used the model to template their fibular lengthening osteotomy and fixation construct [7]. 3D printing has also been shown to be effective in the management of acute foot and ankle trauma. High energy trauma to the foot and ankle can be challenging as anatomical reduction of the articular is crucial for long term success. Zhang et al. presented a cohort of patients who underwent surgical management of high energy ankle fracture dislocations with the assistance of 3D printed models for preoperative planning. They compared this to a cohort of similar patients who did not have preoperative 3D models and found that the patients who underwent fixation with the models had shorter operative times and less intraoperative fluoroscopy and blood loss [8]. Yao et al. similarly created 3D models of calcaneus fractures to assist with preoperative planning but also used the models to pre-countour hardware to ensure it fits appropriately. They found that this technique improved accuracy of hardware positioning and placement and allowed for minimally invasive surgical approaches [9]. 3D printed patient specific cutting guides can be used to ensure precision and accuracy when making bone cuts and osteotomies for deformity correction. Several studies have demonstrated that patient specific instrumentation is accurate and reproducible performing total ankle arthroplasty [10, 11]. 3D printed custom guides have also been designed for subtalar joint arthrodesis, and a recently published study found that these guides reduced operative time and radiation exposure from fluoroscopy [12].

Complex foot and ankle reconstruction is frequently complicated by large osseous defects that require structural bone grafting. Structural grafts typically require significant contouring and can be difficult to mold to the patient’s native anatomy. The graft can also collapse over time which compromises its mechanical integrity. 3D printing has allowed for the development of custom metal implants that provide superior mechanical stability while also conforming to the patient’s anatomy. These custom implants can also be designed with surfaces that promote bone growth and can have areas to pack bone graft. Dekker et al. reported on a cohort of 15 patients who underwent complex lower extremity reconstruction augmented with a 3D printed titanium cage and demonstrated an 87% success rate with 13 of the 15 patients successfully healing their fusion/osteotomy site [13]. Nearly all of the patients in this cohort had a history of previous failed arthrodesis or significant bone loss/deformity from trauma. Reconstructive options for these patients without the assistance of 3D printed technology would be extremely complex and would likely involve large structural allografts and multiple surgeries. Hlad et al. reported on the use of custom 3D titanium implants in the management of bone loss in the setting of failed foot and ankle surgery. They used a titanium cage in cases of a failed total ankle arthroplasty and nonunions of a calcaneal osteotomy and a first tarsometatarsal (TMT) joint arthrodesis. They demonstrated successful healing at 1 year post-op with no complications [14]. 3D printing has revolutionized the treatment of challenging foot and ankle pathology. It allows for better preoperative planning, improved accuracy with bone cuts and osteotomies, and also allows for customized implants in cases of complex deformity and bone loss. The following cases are examples of complex foot and ankle cases in which 3D printing technology was used in surgical management at the author’s institution. The custom metal implants in these cases were designed use the Materialise 3D printing software (Materialise, Plymouth, MI). The implants were printed using the DMP Flex 350 metal 3D printer (3D systems corporation, Rock Hill, SC).

## Clinical applications of 3D printing in complex foot and ankle reconstruction: case series

### Case 1: Tibiotalocalcaneal (TTC) arthrodesis in setting of failed total ankle arthroplasty

**Fig. 1:**
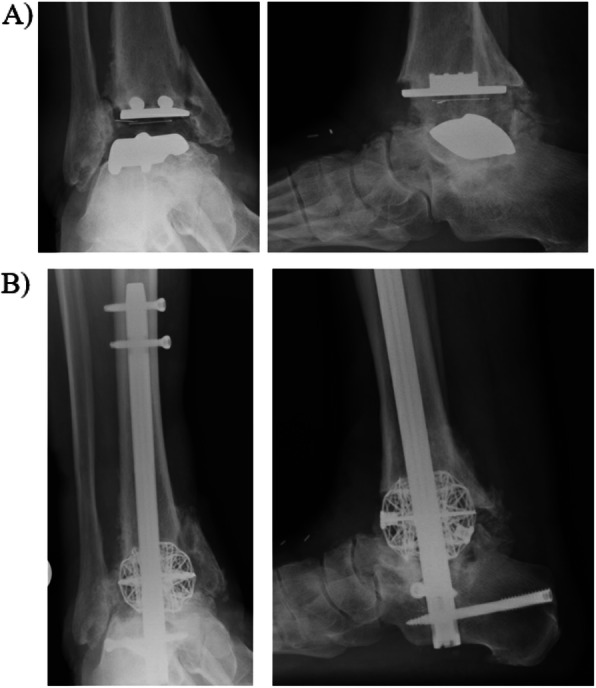
Tibiotalocalcaneal (TTC) arthrodesis for case of failed total ankle arthroplasty. **a** AP and lateral radiographs demonstrate STAR ankle prosthesis with evidence of talar component collapse with erosion into subtalar joint. Medial malleolus fracture present as well. **b** Patient underwent TTC arthrodesis with 3D titanium cage. The cage is packed with allograft/autograft to enhance healing

Failed ankle arthroplasty can be challenging to manage. As talar components collapse, the native talus is eroded away and a large bone defect is often present. These cases can be managed with TTC arthrodesis and bulk structural allograft – most commonly a femoral head. Unfortunately, these complex reconstructions are prone to nonunion (when the bones do not heal together) and the graft can collapse over time. 3D printed cages can serve as augments in these cases to provide structural support and conform to the anatomy of the patient. These cages can be designed to have space for bone grafting and have surfaces designed to improve bony incorporation. Figure 1 is the case of a 65 year old gentleman who presented with a failed total ankle arthroplasty. His talar component had collapsed and eroded through most of the remaining talar bone and into the subtalar joint. He also presented with a medial malleolus fracture. The patient underwent a TTC arthrodesis augmented with a 3D printed titanium cage.

### Case 2: Total talus arthroplasty in the setting of talar avascular necrosis

**Fig. 2:**
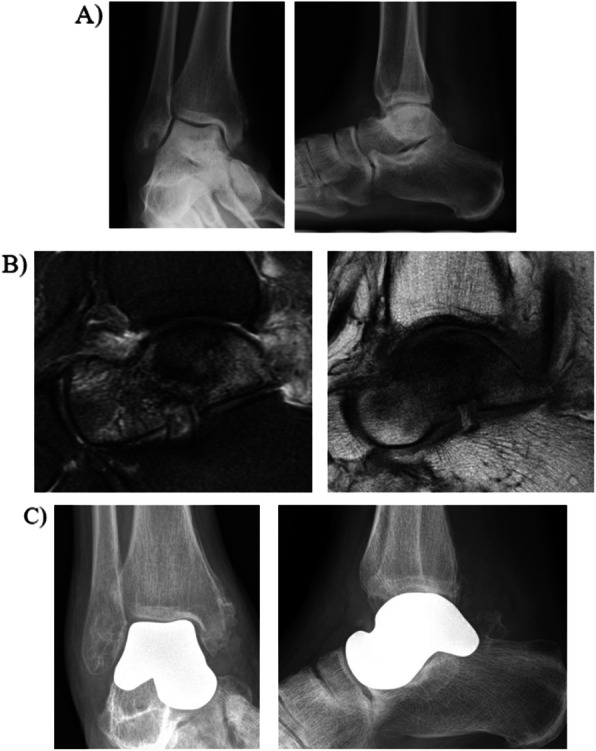
Total talus arthroplasty for talar avascular necrosis. **a** AP and lateral radiographs demonstrate significant sclerosis of the talar body with some central collapse **b** Sagittal T2 and T1 cuts demonstrating diffuse talar avascular necrosis. **c** Total talus arthroplasty with custom 3D printed cobalt chrome prosthesis. Implant is designed based on imaging from the contralateral normal talus

Avascular necrosis of the talus (AVN) is a challenging clinical entity to treat. This disease process occurs when the blood supply to the talus is damaged either by a systemic process or trauma. Nonoperative treatment frequently requires prolonged periods of immobilization which can be detrimental to a patient’s functioning. While early stages of disease can be managed with joint preserving procedures such as core decompression and vascularized bone grafting, advanced disease commonly presents with talar bone collapse. For these advanced cases, prior to 3D printing technology, arthrodesis was routinely the only surgical option, especially with arthritic changes in the ankle or subtalar joint. Like in the previous case, arthrodesis involves removing all avascular bone which leaves a large bone defect. In some instances, talar AVN can present without significant arthritic changes in the surrounding joints. These cases are amenable to total talus arthroplasty with custom 3D printed implants. This implant is designed based on CT images of the talus from the contralateral limb. The implant is made from cobalt chrome and is smooth to allow for gliding at adjacent articulations. Figure 2 represents a case of a 45 year old female who developed talar avascular necrosis in the setting of a previous subchondroplasty. She underwent total talus arthroplasty with a custom 3D printed implant.

### Case 3: Navicular titanium cage in setting of navicular bone loss from ballistic fracture

**Fig. 3:**
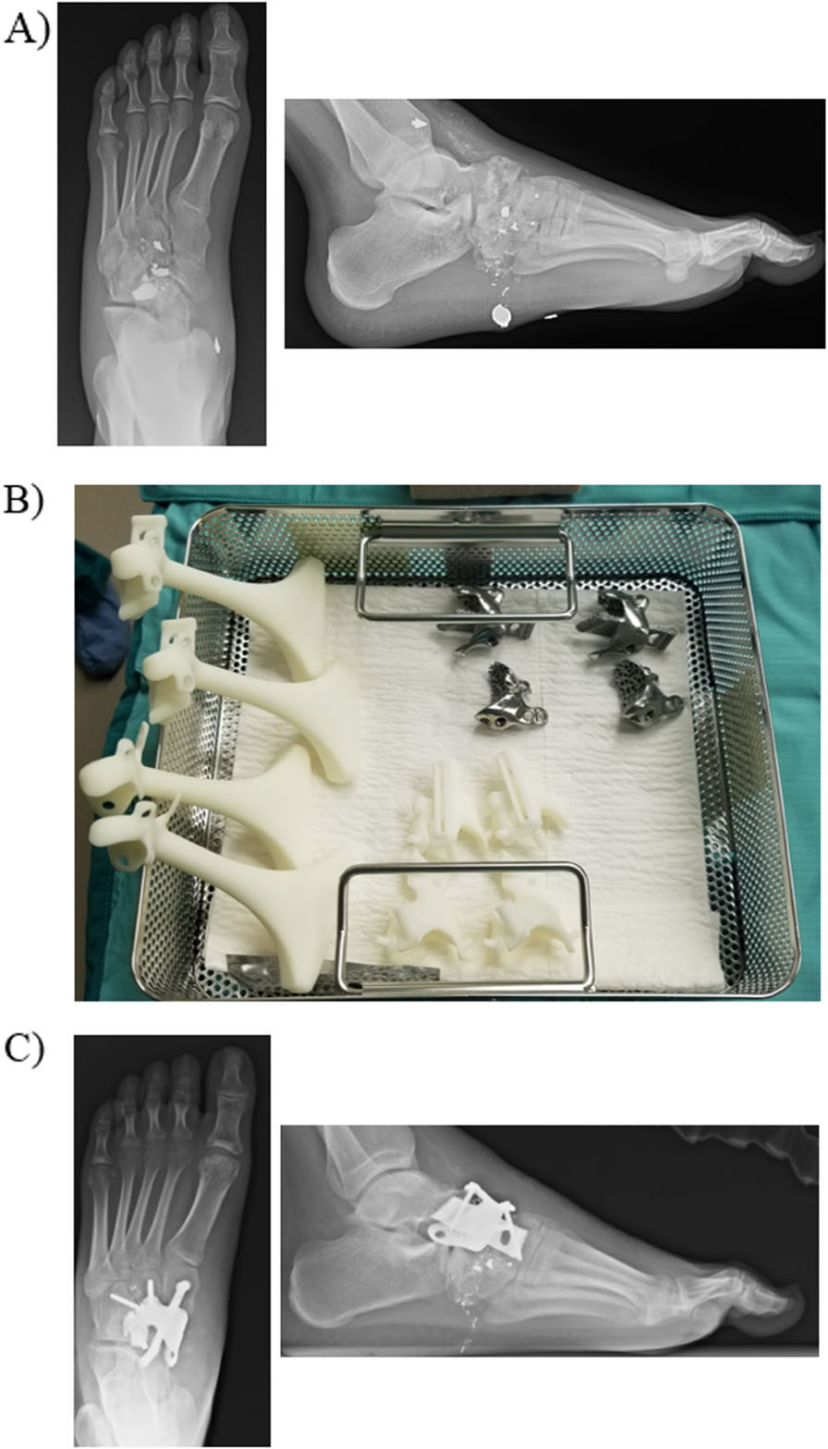
Navicular 3D cage for ballistic navicular fracture. **a** AP and lateral XRs of the foot demonstrating a ballistic comminuted navicular fracture. **b** Sterile operative tray with the 3D printed objects. The plastic objects in the left of the tray are the sizers that are used to determine the implant size that will be used. The bottom of the image shows the custom 3D printed cutting guides. The top contains the 3D printed implants. Multiple sizes are printed and the sizers are used to determine which implant will be used. **c** Immediate postoperative images with the cage construct in place

Ballistic trauma to the foot can be difficult to manage. There injuries typically result in severe comminution making anatomic reconstruction difficult. Ballistic fractures of the navicular can result in shortening of the medial column and this deformity can alter gait biomechanics. Figure 3 is the case of a 23 year old male who sustained a ballistic navicular fracture resulting in severe comminution not amenable to surgical fixation. The patient had a three printed navicular cage designed for a medial column arthrodesis. The implant was designed based on the normal contralateral navicular from a CT scan and built to have struts that would extend out of the navicular cage into the talus and cuneiform to help increase stability. These struts also had bony ingrowth surfaces to promote incorporation. Furthermore, the implant was designed to have multiple possible screws to further enhance stability. In order to ensure the appropriate cuts were made for the struts and the implant, custom cutting guides were also designed to help ensure appropriate fit of the implant.

### Case 4: custom 3D printed cutting guide for a tibial osteotomy

**Fig. 4:**
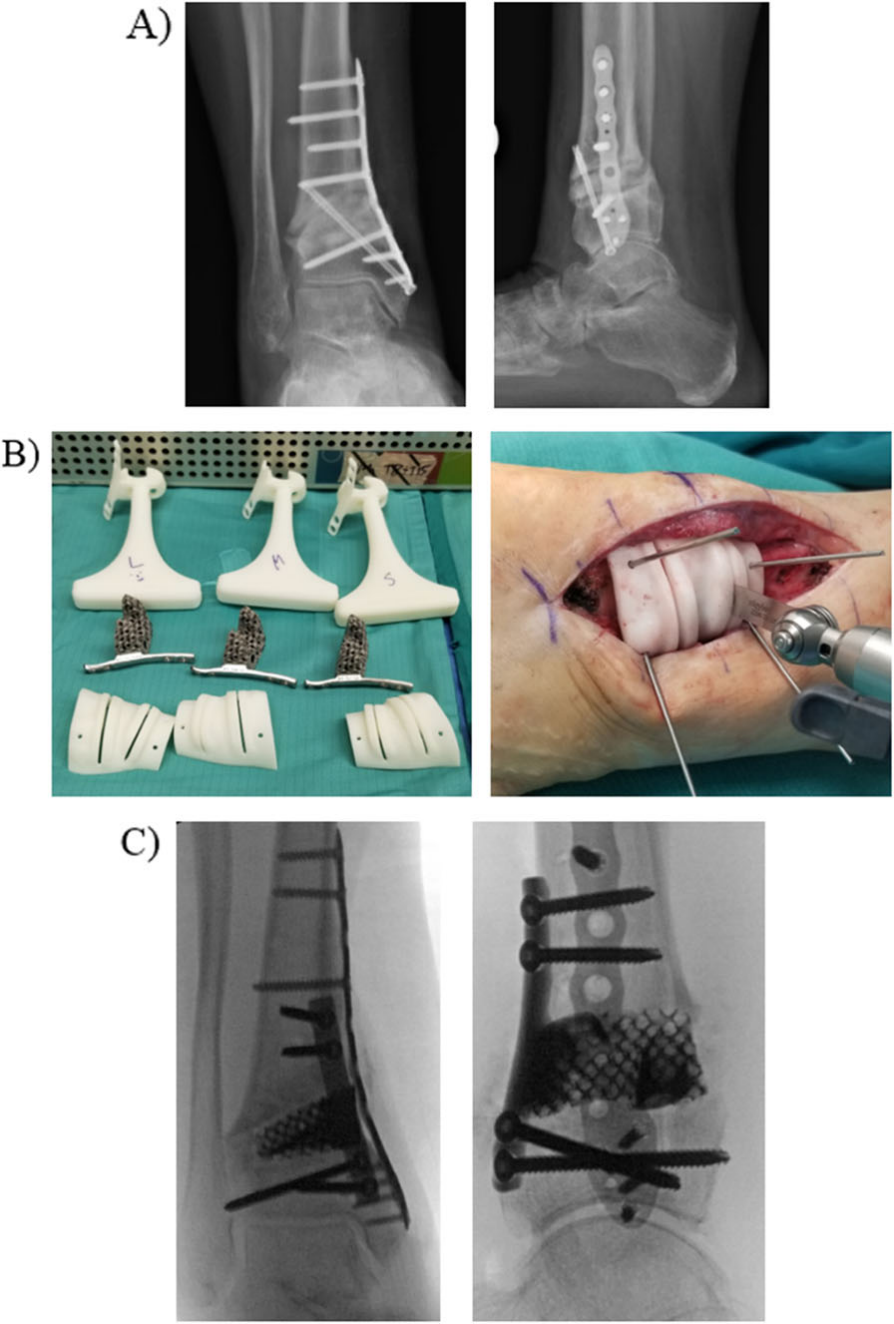
Custom cutting guide for revision tibial osteotomy. **a** AP and lateral views of the ankle demonstrating previous SMO with nonunion. **b** Custom 3D printed sizers, corresponding implants, and custom cutting guides. Cutting guide pinned in place to make appropriate bone cut. **c** Immediate postoperative images with implant in place, bone graft, and additional medial plate added for stability

Angular deformity can be challenging to correct especially when deformity is present in multiple planes. Preoperative planning for these cases is crucial and all planes of deformity must be considered when templating osteotomies and hardware placement. 3D printing technology can be helpful in these cases by providing precise cutting guides to assist with the osteotomies. Figure 4 demonstrates a case that used 3D printed custom cutting guides and implants. This is a 50 year old female who has a history of previous supramalleolar tibial osteotomy (SMO) for a varus deformity that ultimately failed and required a revision surgery. Unfortunately, her revision procedure also went on to a nonunion and she continues to have residual coronal and sagittal plane deformity. She underwent a nonunion takedown and revision distal tibial osteotomy with the assistance of 3D printed custom guides and implants. The implant was designed to fit the patient’s anatomy and correct the deformity. The implant also was printed with a plate attached to it so that fixation could be added directly to the construct.

### Limitations to 3D printing technology

While these cases highlight the versatility of 3D printing within foot and ankle surgery, it is important to understand the limitations that come with this new technology. One of the main drawbacks of using custom 3D printed implants is the cost associated with making the implant. Healthcare costs are a tremendous burden on hospitals and patients – thus use of expensive implants may be denied in favor of more traditional and cheaper implants. However, as the technology continues to improve, costs of production will decrease and make these implants more affordable. The time it takes to design and manufacture the implant is also a limitation and it can take at a minimum four to 6 weeks for an implant to be made. This time delay has functional and economic consequences to the patient who continues to have pain and may be unable to work. It is important to note that this four to 6 week time frame is from experience at our institution and may vary between locations and practices. Finally, the technology is new thus there is a learning curve associated with its use. Each case is unique and presents its own challenges which adds complexity to using a custom implant and instrumentation. Surgeons must take extra time to prepare for each case and inspect the instruments and hardware before the case begins to better anticipate any intraoperative difficulties that may arise with its use.

## Conclusion

3D printing technology has revolutionized the manufacturing industry. As the technology has advanced over the past several years, its clinical utility and applications have also increased. 3D printing in orthopaedic surgery can be used to improve preoperative planning, customize implants and instruments, and improve surgeon education and training. Within foot and ankle surgery, orthopaedic surgeons can use 3D printing technology in the surgical management of complex deformity and cases of significant bone loss.

## Data Availability

Not applicable.
